# Acute urinary retention due to benign prostatic hyperplasia associated with cystitis glandularis in a 22-year-old patient

**DOI:** 10.11604/pamj.2018.30.30.14835

**Published:** 2018-05-16

**Authors:** Skander Zouari, Khaireddine Bouassida, Khaled Ben Ahmed, Ahlem Bdioui Thabet, Mohamed Amine Krichene, Chawki Jebali

**Affiliations:** 1 Urology Department, Sahloul Hospital, Sousse, Tunisia; 2 Histopathological Department, Farhat Hached Hospital, Sousse, Tunisia; 3 Radiology Department, Sahloul Hospital, Sousse, Tunisia; 4 Emergency Department, Sahloul Hospital, Sousse, Tunisia

**Keywords:** Benign prostate, hyperplasia, young male, cystitis glandularis, dysuria

## Abstract

A 22-year-old man has consulted in emergency for acute urinary retention and left renal colic. Bladder catheterization was performed. Symptomatic treatment was provided with no improvement. MRI showed a pseudotumoral bladder wall thickening associated with vesical floor budding with prostate median lobe infiltration. The patient got an endoscopy that concluded to an inflammatory aspect of the bladder mycosa and a solid mass in the bladder neck arising. The biopsy during examination concluded to a glandular cystitis. Ultrasonography performed six months later still showed an enlarged prostate of 60g volume, post void residue of 280ml and bilateral hydronephrosis. A second cystoscopy showed an obstructive prostate with a median lobe. A transurethral resection of this lobe was performed. The pathological examination concluded to a benign prostate hyperplasia. This case is likely to be the first reported so far about a BPH in a young male associated with Cystitis Glandularis. Neither etiology nor evidence of the cause behind this case has been identified so far. Although Benign Prostate Hypertrophia is rare among young males, its ethiopathogenesis is not well known, its relation with cystitis glanduralis in young patients has never been described before. Both medical and surgical approaches remain similar to the adults.

## Introduction

Benign prostatic hyperplasia (BPH) is a common disease that usually develops in middle-aged men. It is very rare in the third decade of life, around 7cases have been reported so far and no cases of associated BPH and cystitis glandularis has been reported so far in young patient.

## Patient and observation

**Figure 1:**
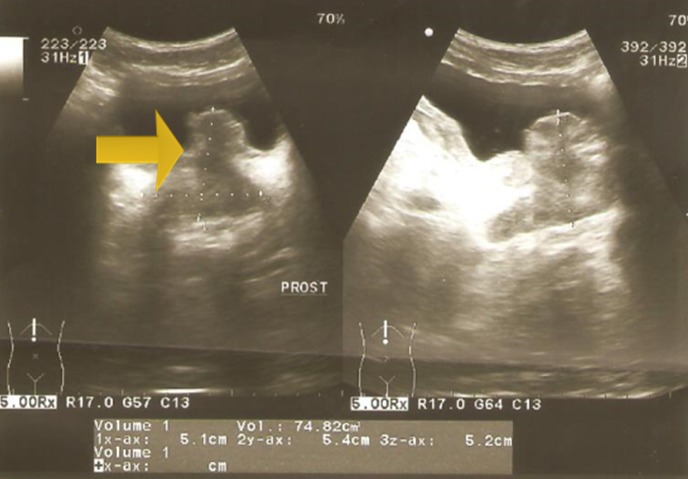
Bladder wall thickeness

**Figure 2:**
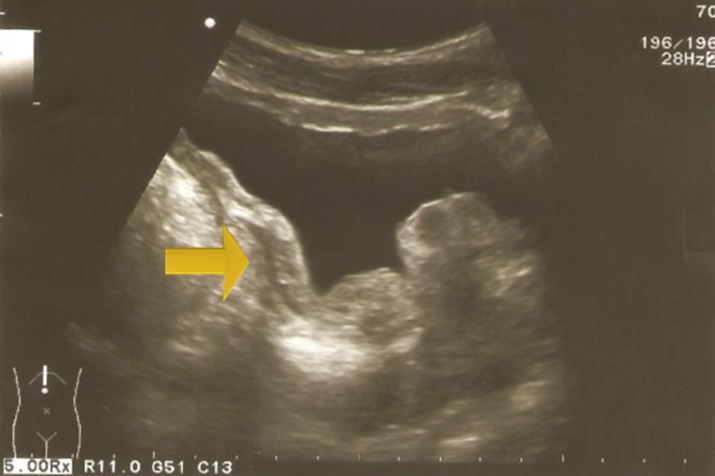
Enlarged prostate of 60 gr

**Figure 3:**
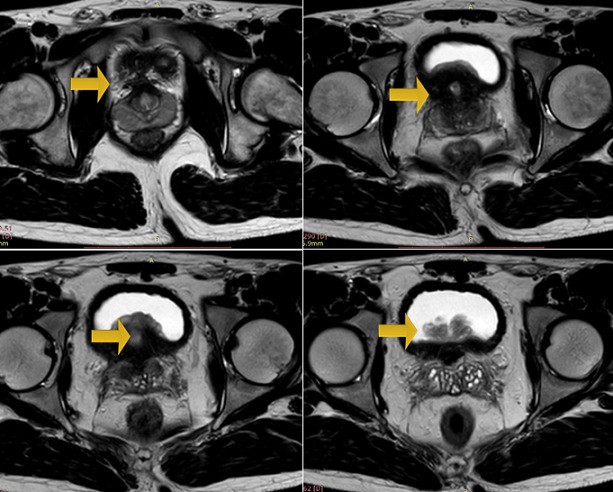
Axial T2 weighted sequence showed an enlarged prostate gland associated with a lobulated arising from the cystic floor

**Figure 4:**
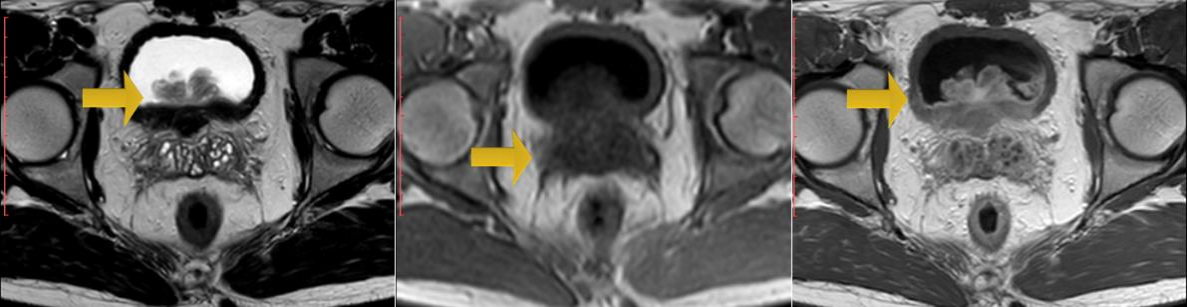
The bladder floor mass presents as a low signal mass on T1, heterogenious signal on T2 with central branching hyperintensity.This central hyperintensity showed an avid enhancement on contrast administration and represents the vascular stalk. These findindings, are evocative of Cystitis glandularis

**Figure 5:**
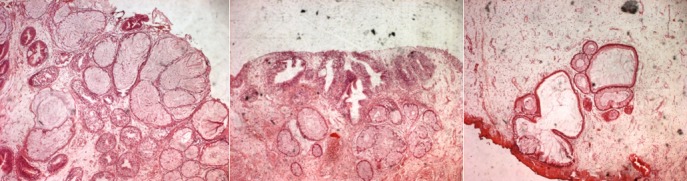
The lesions consisted of a proliferation of glands in the lamina propria, lined by columnar epithelium, with mucin production. There was no significant nuclear hyperchromasia, pleomorphism. (H-E x 40)

**Figure 6:**
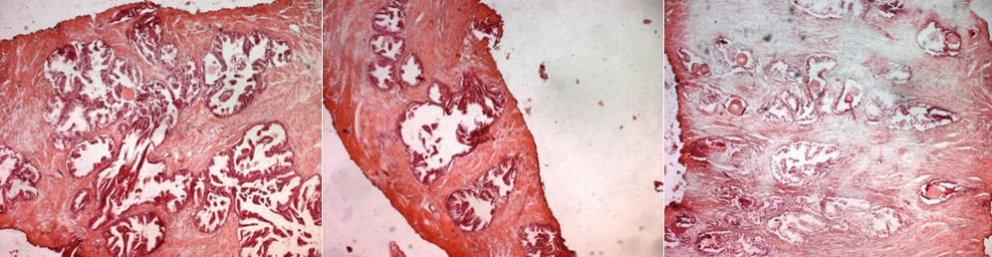
Benign prostatic hyperplasia involving both glands and stroma. The hyperplasicglands are, well-differentiated, crowded, separated by stroma, with corpora amylacea in the lumens (H-E x 40)

**Figure 7:**
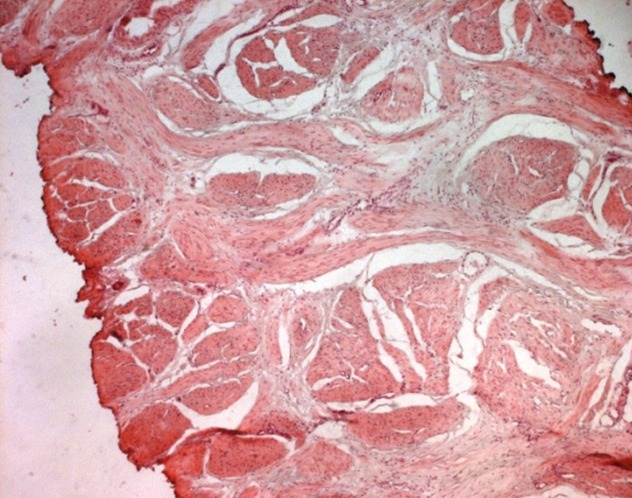
Low power view of an area of stromal hyperplasia with bundles of smooth muscle. (H-E x 40)

A 22-year-old man, with no past medical history, has consulted in emergency for acute urinary retention and left renal colic. Bladder catheterization was performed as well as an ultrasound that showed left obstructive premeatic calculi of 6mm and bladder distension. On examination the patient was afebrile and a slight tenderness on the left flank was found on abdominal palpation. On DRE we found an enlarged prostate, there were no indurations and no areas of softness or tenderness. An anti-inflammatory and antalgic treatment was provided to the patient. After one week we take off the bladder catheterization but the patient still presents a dysuria. Another ultrasound was performed; there was an inflammatory aspect of the bladder with a thickened bladder wall and a tissular proliferation measuring 5*2 cm on the bladder neck and at the left wall with large implantation and probably prostate infiltration. It was vascularized on color Doppler ultrasound (Figure 1, Figure 2). Meanwhile, the patient presented another episode of acute urinary retention few weeks later. Biological exams were normal. Urine Analysis was normal. PSA was about 1.6 ng/ml. The MRI showed a pseudotumoral bladder wall thickening associated with vesical floor budding with prostate median lobe infiltration (Figure 3, Figure 4). The patient was admitted for an endoscopic examination. It was performed on March, 9th, 2016, finding an inflammatory aspect of the bladder mycosa and a solid mass in the bladder neck arising from the prostate most likely. It was hard to distinguish its origin, whether it is from the prostate or from the bladder. A cystoscopy was performed and a biopsy of the presumed mass was done. The pathological examination concluded to a glandular cystitis without any indicative of malignancy (Figure 5). In the follow up the patient still has dysuria and urinary leak. The ultrasound performed six months later showed an enlarged prostate of 60gr volume, post void residue of 280ml and bilateral hydronephrosis. The patient was hospitalized again in March 2017 and a second cystoscopy was performed. The endoscopic examination showed that the prostate was obstructive with a median lobe. A trans urethral resection of this lobe was performed. The pathological examination concluded that it was a benign prostate hyperplasia (Figure 6, Figure 7).

## Discussion

The average prostate volume from the age of 1 to 10 is 1.4cc (0.4g) and 10.8cc (3.8g) from 11 to 20. From 21 to 30, it is 19.8cc [1,2]. Xia and al [3] described four phases of the prostate growth, first slow phase (0 to 9 years), first rapid phase ( from 10 to 30 years), second slow phase (from 30 to 50 years), and second rapid phase (from 50 to 90 years). According to ultrasonographic findings in their study, the prostate volume was approximately 15 ml at the end of childhood. Up to date, only few cases about benign prostate hyperplasia among young men have been reported so far. Some authors explained the possible etiologic factors of their cases e.g: Powell [4] reported a case of a 17-year-old patient who developed benign prostatic hyperplasia. He speculated that it was caused by gonadotrophin administration for the treatment of cryptorchidism. Sumiya et al. [5] reported a case of a 20-year-old patient whose 380-g prostate was removed by suprapubic prostatectomy; however, the cause was not described. Romano and al. [6] reported a case of a 23-year-old man who presented an acute urinary retention revealing a BPH. Romano studied the relation between BPH and acromegaly due to the height of the patient (2.03m). Choi and al. [7] described a case of a10 year-old boy with hydroureteronephrosis with a prostate of 30 gr. In this case, Choi has studied the effect of taking human chorionic gonadotrophin-containing agent during pregnancy to prevent spontaneous abortion and its synergic effect with the androgen in prostate hyperplasia. Fanourios G et al. [8] reported a case of a 27-year-old man whith BPH, a 181 g prostate volume revealed by Low urinary tract symptoms and hemospermia. Again, the cause of the BPH was not described. Our patient also had no history of drug intake, endocrinologic disease, or any other possible relevant factors.

However, BPH was associated to cystitis glandularis with an unusual proliferative disorder of the urinary bladder, which is characterized by transitional cells that have undergone glandular metaplasia [9]. Its potential premalignant significance is still the subject of debate. Although it was suggested that cystitis glandularis should be considered as a precancerous lesion [10], some other studies found no concrete evidence in its role in developing bladder carcinoma [11]. In addition, Cystitis glandularis is infrequent in young people and has rarely been reported [12]. However, the exact incidence of cystitis glandularis is nonetheless unknown. To our knowledge no case of associated BPH and glandular cystitis in young patients was so far reported. These unusual cases suggest other possible etiologic factors or circumstances are involved in BPH, and more data are needed. BPH is commonly revealed by LUTS, and in our case, our patient was admitted with acute urinary retention. Acute urinary retention in young people related to a wide spectrum of diseases including neuropathic processes, severe voiding dysfunction, constipation, urinary infection, urolithiasis, and tumor [13]. After performing urine catheterization, imaging is essential for the investigation. The DRE is necessary and can find a mass that could be a rhabdomyosarcoma or a rare case of BPH. Ultrasonography, CT scan and MRI could evaluate the mass detected in the DRE. Despite all the information provided by the clinical exam and imaging, the endoscopic examination and biopsy for pathological examination remains necessary to determine the diagnosis. After confirmation, trans urethral resection of the prostate could be performed. The treatment of the BPH in adults consists of medical and surgical approaches. Medical treatment is based on alpha blockers and/or 5-alpha reductase inhibitors. In case of failure of medical treatment, or complications such as urinary acute retention, urinary infections, or bladder lithiasis, endoscopic surgery or open prostatectomy can be performed depending on the prostate volume. However, the safety of long-term use of these drugs is unknown in the children and young people. Due to the small number of cases of BPH in young people, there is no consensus established in the treatment. Long term follow up is mandatory for detecting long term complications and for the unknown nature of the disease in young people. Last time, our patient has consulted on June, 5^th^, 2017. He did not report any complaints, nor voiding troubles or sexual ones so far.

## Conclusion

Although benign prostate hypertrophia is rare among young males, its ethiopathogenesis is not well known. Some hypothesis have been studied as causing BPH, but their relation with cystitis glanduralis in young patients has not been described before, and the cause remain unknown. There is no specific treatment for young males for BPH and for Cystitis, and both medical and surgical approaches remain similar for adults.

## Competing interests

The authors declare no competing interests.
